# Abstinent Heroin Addicts Tend to Take Risks: ERP and Source Localization

**DOI:** 10.3389/fnins.2017.00681

**Published:** 2017-12-06

**Authors:** Qinglin Zhao, Hongqian Li, Bin Hu, Haiyan Wu, Quanying Liu

**Affiliations:** ^1^Ubiquitous Awareness and Intelligent Solutions Lab, Lanzhou University, Lanzhou, China; ^2^Institute of Psychology, Chinese Academy of Sciences, Beijing, China; ^3^Department of Psychology, University of Chinese Academy of Sciences, Beijing, China; ^4^Movement Control & Neuroplasticity Research Group, KU Leuven, Leuven, Belgium; ^5^Neural Control of Movement Laboratory, ETH Zurich, Zurich, Switzerland

**Keywords:** gambling task, decision making, ERP, stimulus preceding negativity, feedback related negativity, P300, source localization, heroin addiction

## Abstract

Abnormal decision making is a behavioral characteristic of drug addiction. Indeed, drug addicts prefer immediate rewards at the expense of future interests. Assessing the neurocognitive basis of decision-making related to drug dependence, combining event-related potential (ERP) analysis and source localization techniques, may provide new insights into understanding decision-making deficits in drug addicts and further guide withdrawal treatment. In this study, EEG was performed in 20 abstinent heroin addicts (AHAs) and 20 age-, education- and gender-matched healthy controls (HCs) while they participated in a simple two-choice gambling task (99 vs. 9). Our behavioral results showed that AHAs tend to select higher-risk choices compared with HCs (i.e., more “99” choices than “9”). ERP results showed that right hemisphere preponderance of stimulus-preceding negativity was disrupted in AHAs, but not in HCs. Feedback-related negativity of difference wave was higher in AHAs than HCs, with the P300 amplitude associated with risk magnitude and valence. Using source localization that allows identification of abnormal brain activity in consequential cognitive stages, including the reward expectation and outcome evaluation stages, we found abnormalities in both behavioral and neural responses on gambling in AHAs. Taken together, our findings suggest AHAs have risk-prone tendency and dysfunction in adaptive decision making, since they continue to choose risky options even after accruing considerable negative scores, and fail to shift to a safer strategy to avoid risk. Such abnormal decision-making bias to risk and immediate reward seeking may be accompanied by abnormal reward expectation and evaluation in AHAs, which explains their high risk-seeking and impulsivity.

## Introduction

“Drug addiction is a chronic relapsing disorder characterized by a compulsion to seek and take drugs, loss of control in limiting intake, and the emergence of a negative emotional state in the absence of drug” (Koob, 2005). It largely affects cognitive functions, which could also be reflected in decision making tasks (Rogers et al., 1999), e.g., delay discounting procedure (DDP), gambling task (GT), and ultimatum game (UG) (Vassileva et al., 2007; Hou et al., 2016). Drug addicts often show abnormal decision-making behaviors and prefer to seek for short-term interests and ignore long-term consequences in risky decision-making tasks (Higley and Bennett, 1999; Tarter et al., 2003; Fishbein D. H. et al., 2005). Besides, they hardly avoid imminent threats or negative consequences by shifting to a safer strategy, which helps prevent from penalty or increase the likelihood of reward (Dunn et al., 2006). Typically, such abnormal decision making is associated with several possible factors, including cognitive impulsivity, delay in aversion, and hypersensitivity to reward (Must, 2008) or hyposensitivity to punishment in drug-dependents patients (Bechara et al., 2002; Bechara, 2005; Everitt et al., 2008; Hanson et al., 2008). According to previous studies, abnormal decision-making is associated with brain areas such as the ventral medial prefrontal cortex (vmPFC) (Boes et al., 2009), anterior cingulate cortex (ACC) (Rogers et al., 2004; Fishbein D. H. et al., 2005) and amygdala (Clark and Robbins, 2002). However, the neural correlates of abnormal risk decision making in abstinent heroin addicts (AHAs) remain unclear. Assessing behavioral and neural evidences in decision making tasks in drug-dependents patients may contribute to finding common mechanisms related to addiction behavior (e.g., higher sensitivity to reward-related stimuli or punishment stimuli) and its development (e.g., self-administrated drug-seeking, withdrawal, and relapse).

The gambling task has been widely used to examine abnormal decision-making behavior in addicted subjects (Ersche et al., 2005; Fishbein D. et al., 2005). Previous studies have shown that cocaine (Grant et al., 2000), alcohol (Mazas et al., 2000; Gungor et al., 2014) and internet (Seok et al., 2015) addicts perform differently compared with healthy controls in some decision-related gambling tasks such as the Iowa Gambling Task (IGT) and Cambridge Gamble Task (CGT). Neuroimaging studies have also assessed the neural mechanism of risky decision-making in IGT by positron emission tomography (PET) (Tanabe et al., 2007) and functional magnetic resonance imaging (fMRI) (Kircheis et al., 2004; Sohrabi et al., 2015). Similar to IGT, CGT was developed by Rogers et al. (1999) to assess decision-making disorders in patients by analyzing the orbital prefrontal cortex, while evaluating patients for risk-taking behavior under decision-making tasks. Although IGT and other gambling tasks are widely used in addiction studies, criticisms have been raised over both their designs and data interpretations (Dunn et al., 2006). Therefore, in the current study, we adopted a simple and easy-to interpret gambling task (two-option task) developed by the IGT, to examine the abnormal risky decision-making behavior of AHAs in anticipation and outcome evaluation (i.e., both reward and punishment processing).

Regarding decision-making deficits in heroin addicts, multiple studies have reported abnormal decision-making (Petry et al., 1998), which was also observed in AHAs (Rogers et al., 1999). However, the neural correlates of abnormal decision-making in relation to drug dependence remain unknown. We hypothesized that abnormal sensitivity of reward and punishment in AHAs, which are driven by the impulsive and reflective brain systems, may lead to risk-seeking behaviors in AHAs (Bechara, 2005). The event-related potential (ERP) technique, which helps assess the behavioral-neuronal relationship between the two regions with high temporal resolution (Pires et al., 2014), is an effective measure of neural activity and may contribute to testing our hypothesis of the underlying mechanism of decision making abnormality in AHAs. A few existing studies have related ERPs with cognitive processing state during decision-making tasks. In the current study, we specifically focused on key ERP components and demonstrated their associations with reward/punishment expectation and reward/punishment evaluation, e.g., stimulus-preceding negativity (SPN), feedback-related negativity (FRN), and P300.

The pre-feedback SPN is a slow, right-hemisphere dominant negativity with frontal distribution, and is considered an index of expectation during reinforcement learning (Brunia et al., 2011). SPN is typically observed in the waiting period of the outcome, expressing motivational/attentional engagement due to possible informative or emotionally relevant feedback (Morís et al., 2013). It is thought to be mainly generated by the anterior insular cortex (Brunia et al., 2000). Previous studies have indicated enhanced SPN for addiction cues. For instance, expectation of smoking related cue elicits larger SPN for smokers than nonsmokers (Parker and Gilbert, 2008). Considering the hypothesis of hypersensitivity to reward/punishment hypothesis in AHAs, we expect that the SPN amplitude in AHAs would be larger due to the enhanced affective-motivational anticipation of outcomes.

In the outcome feedback stage, the impact of reward delivery was evaluated by the amplitude of FRN, a negative-going deflection maximal at fronto-central scalp electrode sites around 250 ms post-feedback, and P300, a positive-going deflection maximal more parietal around 300–500 ms. FRN and P300 encode different aspects of outcomes. On the one hand, FRN is sensitive to reward valence (gain or loss) and expectancy toward outcomes with the inconsistency of expectancy eliciting more negative-going FRN (Zhou et al., 2010). Previous fMRI studies suggested that FRN is generated in the medial frontal cortex, and probably in the ACC (Amiez et al., 2005). Moreover, FRN is associated with reinforcement learning and punishment sensitivity. The reinforcement learning system is capable of rapidly determining whether a feedback is better or worse than expected, and encoding the difference between expectations and actual outcomes as a reward prediction error (Tzur and Berger, 2009). Based on detection of these prediction errors, ACC plays an important role in the top-down cognitive control (Botvinick et al., 2004; Barber and Carter, 2005). On the other hand, P300 is discussed in terms of relatively high-level evaluation of feedback (Li et al., 2010). Previous studies have shown that the reward P300 is sensitive to various aspects of outcomes, including reward magnitude (Nittono et al., 2008) and reward valence (Yeung et al., 2005; Wu and Zhou, 2009). We expect a decreased pre-feedback SPN amplitude in the right hemisphere and an enhanced FRN difference wave in AHAs compared with HCs. The amplitude of FRN difference wave impacted by immediate rewards would be higher in AHAs than HCs. In addition, whether P300 is associated with valence (gain or loss) and magnitude (high- or low-risk) will be assessed. In this case, ERPs might be a potential technique to evaluate neural correlates of mental process in different stages of risky decision-making in AHAs. Furthermore, source localization may help examine the neuronal activation during the decision-making processes at the gambling task. The abnormal decision-making behaviors of AHAs may be caused by a weakened impulse-control system (e.g., cognitive frontal- and affective systems) during the gambling task (Chaiken and Trope, 1999; Simon and Daw, 2012). We assumed that a weakened reflective and insula mediated neural system may influence the risky decision-making behavior of AHAs. However, the low temporal resolution of fMRI does not allow the disentanglement of the rapid cognitive processes underlying decision making. Combined with source imaging based on multiple channel EEG signals, we were interested in exploring the associated brain regions involved in sequential cognitive states during a gambling task.

In the current study, we extracted ERP components associated with reward expectation and outcome evaluation stages to assess the functional substrates in the decision-making process during the gambling task. Controlling expectation value and probabilities, we mainly observed reward expectation and outcome evaluation by the risk of decision-making (high- vs. low-risk) and reward valence (win vs. loss). Based on previous neurobehavioral evidence (Bolla et al., 2003), we predicted that performance on a gambling task in AHAs would show a tendency toward greater risk-taking and less sensitivity to punishment, particularly at the higher level of risk and potential reward magnitude. Considering the insula is connected to widespread regions of the prefrontal cortex that integrate interception states into conscious feelings and decision-making processes involved in uncertain risk and reward, we also predicted that the reflective and insula mediated neural system accounts for decision-making deficits (i.e. prioritizing short-term consequences of a decisional option), leading to elevated addiction risk and relapse.

## Materials and methods

### Participants

Forty participants were involved in the current study, including 20 AHAs (aged 35.40 ± 8.18 years; 10 females and 10 males) and 20 HCs (aged 31.80 ± 5.72 years; 10 females and 10 males). AHAs were recruited from the Gansu Compulsory Isolated Detoxification Center in China, meeting the criteria of Diagnosis and Statistics of Mental Disorder 5th edition (DSM-V) for heroin dependence. The average duration of drug abuse and weekly dosage of AHAs were 6.56 ± 4.54 years and 3.20 ± 2.25 g, respectively. The AHAs who participated in our study were abstinent from heroin and other dependent drugs for at least 4 month (abstinent period: 9.00 ± 3.36 months). HCs were recruited from the local community, and had no history of alcohol or drug abuse. These two groups showed no significant differences in age [*t*_(38)_ = 1.613, *p* = 0.115] or education level [AHA, 9.55 ± 2.26 years; HC, 8.80 ± 2.505 years; *t*_(38)_ = 0.994, *p* = 0.326]. All subjects were right-handed, with normal or corrected-to-normal visual acuity, with no history of neurological problems. None of the subjects was taking any psychotropic, neurological, or psychiatric medications at the time of experiment. Potential participants were excluded when they had any history of other addictions or any neurological or psychological disorders. All participants provided written informed consent before enrolment in the study, which was approved by the Ethics Commission of Institute of Psychology of the Chinese Academy of Sciences (*Approval Number: H15020*).

### Stimuli and task

**Figure 1:**
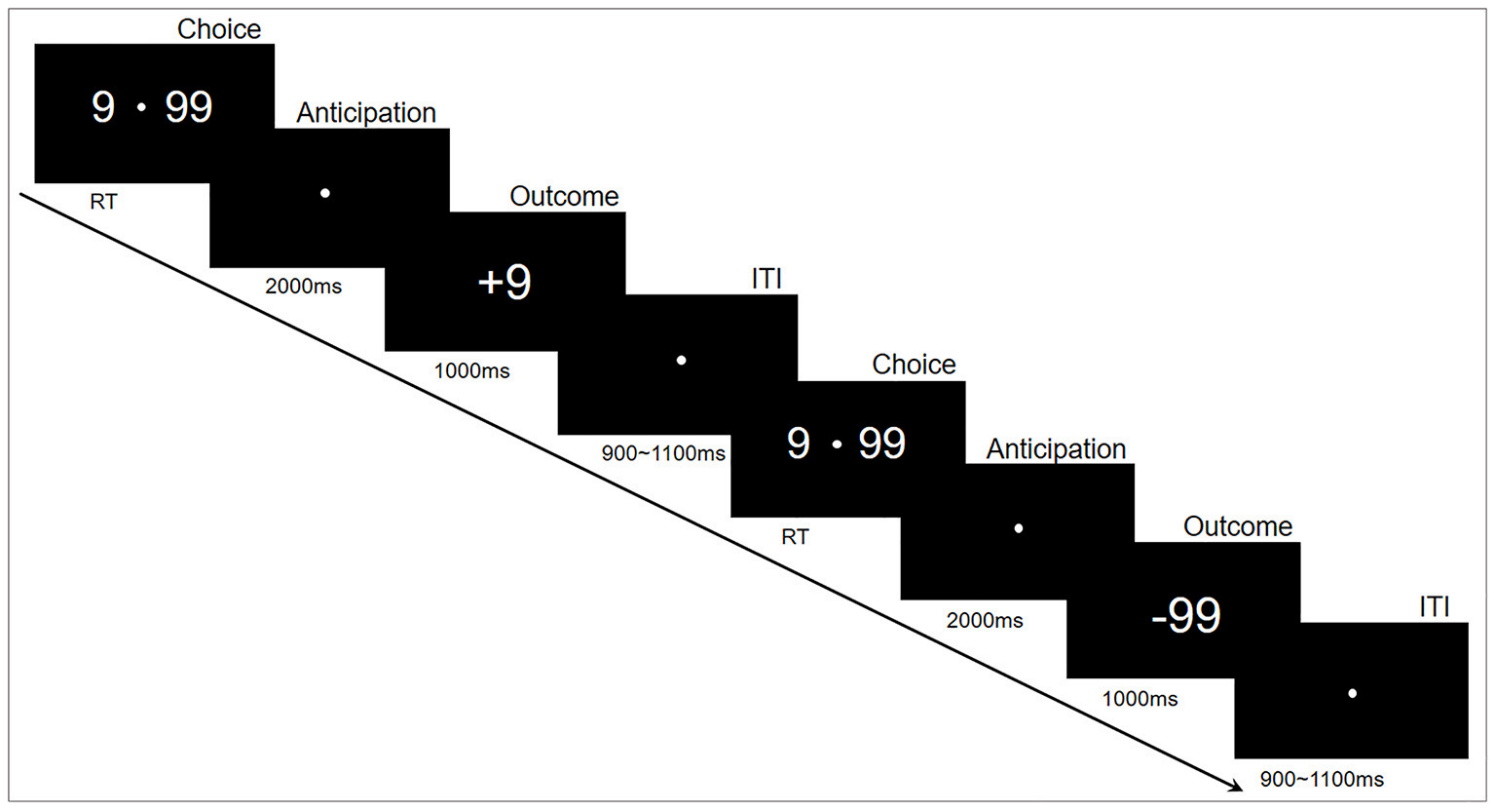
Experimental paradigm. Each trial began with two options (number “9” and “99”) that appeared on either side of a fixation point. The participants then selected one of the two alternatives by pressing a button. Following their responses, a fixation point was presented in the center of the monitor for 2,000 ms; thereafter, a positive or negative number appeared for 1,000 ms. Each trial ended with an intertribal interval (ITI) varying randomly from 900 to 1,100 ms.

The experiments were performed in accordance with approved guidelines, in a quiet, air-conditioned laboratory with dim natural light. The participants sat comfortably at 80 centimeters in front of a 21-inch computer monitor. The gambling task was programmed and presented using E-prime2.0 (Psychology Software Tools, Inc.). The experimental paradigm is shown in Figure 1. Specifically, each trial began with two options (“99” and “9”) presented on either side of a fixation point in the center of the monitor. The participants then selected one of the two alternatives by pressing a button as soon as possible, corresponding to the location of the selected option, with either their left or right index finger (“F” or “J”). This pair of options remained on the monitor until a choice was made. Following their responses, the fixation point remained in the center of the monitor for 2,000 ms while the options disappeared. Finally, the outcomes (“+99” or “−99” for the choice of “99”, “+9” or “−9” for the choice of “9”) were displayed in the center of the monitor for 1,000 ms to indicate the gain or loss for this trial. The inter-trial interval was varied randomly from 900 to 1,100 ms. The whole task consisted of 400 trials in five blocks of 80 trials that lasted about 6 min each. The subjects had a practice block with 10 practice trials before the formal experiment blocks; a short break of about 1–2 min was inserted between blocks. To reduce excessive eye movements and blinks, the participants were instructed to keep fixation on a white point in the center of the monitor during the experiment.

This experiment was designed to assess the risky decision-making behavior. The option “9” was defined as low-risk choice that would yield either a gain or loss of 9 credits, whereas “99” was the high-risk option (high variation) that would yield either a gain or loss of 99 credits (Zheng and Liu, 2015). The probabilities of the gain or loss for each option were identical; therefore, the expectation of final credits was zero. The participants were encouraged to use any strategy to maximize the sum of credits. The higher credits they collected in the task, the more money they would win in the end.

### EEG recording and processing procedures

EEG signals were recorded during the task using a 64-channel electrode cap (Brain Products, Gilching, Germany) with the International 10/20 System sites. The scalp impedance of each sensor was kept below 10 kΩ. EEG signals were recorded at a sampling rate of 1,000 Hz with the vertex as a reference, and amplified with an analog band-pass filter of 0.01–100 Hz. According to the quality of EEG signals, 2 AHAs and 3 HCs were excluded, and 20 AHAs sand 20 HCs were included in the final analysis.

EEG data were processed with Brain Vision Analyzer 2.0 (Brain Products, Gilching, Germany) using following steps. First, raw EEG signals were re-referenced offline to the mean of activity at the left and right mastoid electrodes (TP9/TP10) (Liu et al., 2015). Then, independent component analysis (ICA) was performed to remove ocular and muscle artifacts. Afterward, the artifact-removed EEG signals were band-pass filtered: 0.1–20 Hz for SPN analysis, and 1–30 Hz for FRN and P300 analyses, to minimize possible interferences from SPN (Zheng and Liu, 2015). Two types of EEG signals were finally segmented into epochs that were time-locked to the feedback onset. Based on our experimental design, SPN was averaged from the epochs between 1,800 ms prior to and end 200 ms post feedback onset, with activity from −1,800 to −1,600 ms serving as baseline. FRN and P300 were epoched between 100 ms pre-feedback and 1,000 ms post-feedback, with activity from −100 to 0 ms serving as baseline. Moreover, EEG epochs with absolute voltage exceeding 100 μV were automatically excluded from the analysis. To enhance the signal to noise ratio, attempts to superimpose the ERP waveforms were no less than 30 for each condition.

### Analysis of ERP components

**Figure 2:**
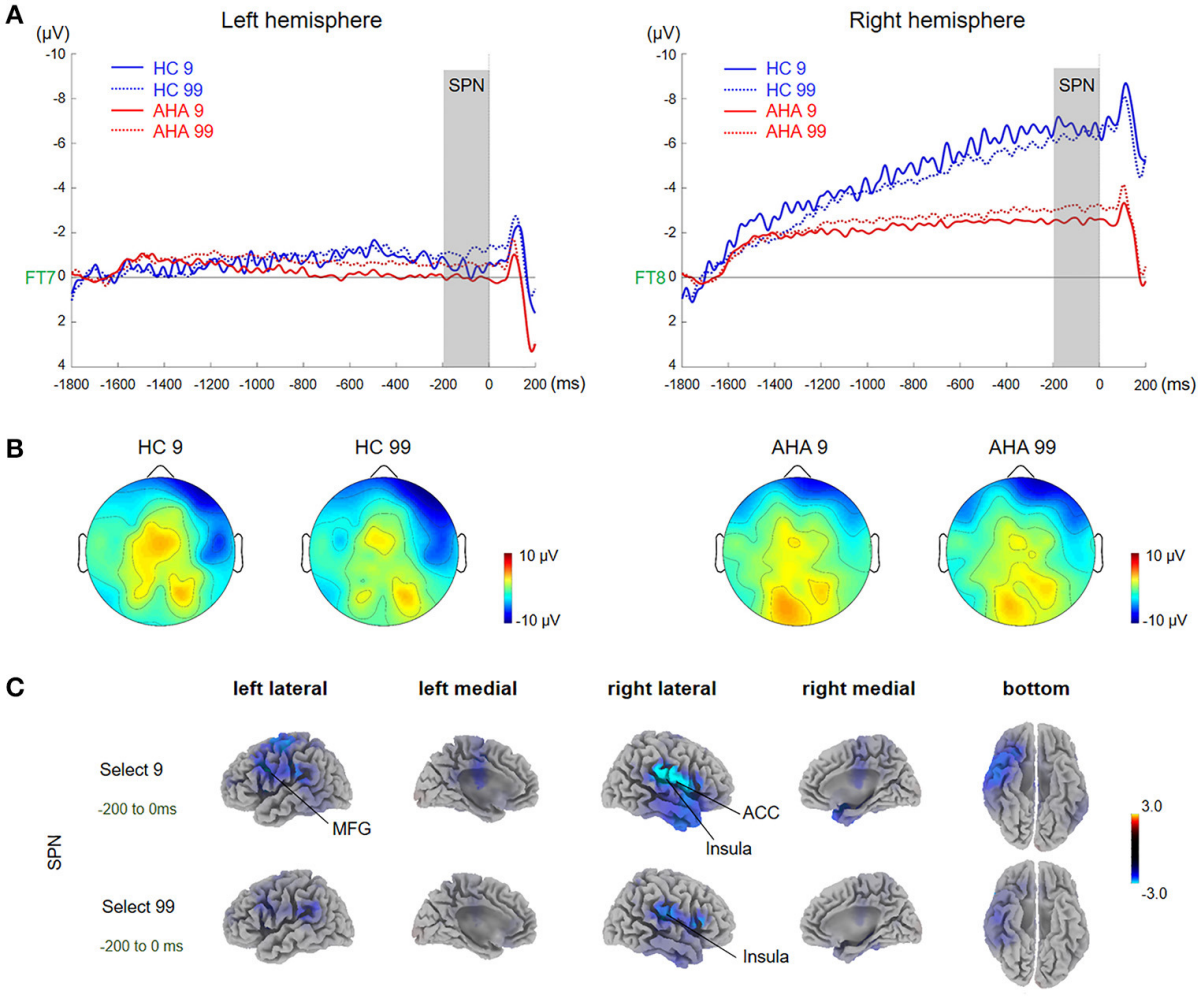
Waveforms and scalp topographies of SPN. **(A)** Grand average ERP waveforms following low- and high-risk decision-making for AHAs and HCs at FT7 and FT8; the shaded areas depict the time window of stimulus-preceding negativity (SPN). **(B)** Topographic maps of the mean amplitude of SPN (−100 to 0 ms). **(C)** The *t*-score maps for comparing AHAs and HCs for the SPN component for low- and high-risk options. The brain regions labeled with yellow/red indicate AHAs > HCs, whereas green/blue indicates AHAs < HCs. The time periods for SPN are also indicated. Significant brain regions (*p* < 0.05, FWE corrected) are shown on the map. The peak MNI coordinate regions for each comparison are reported in **Table 1**. ACC, anterior cingulate cortex; MFG, middle frontal gyrus.

**Figure 3:**
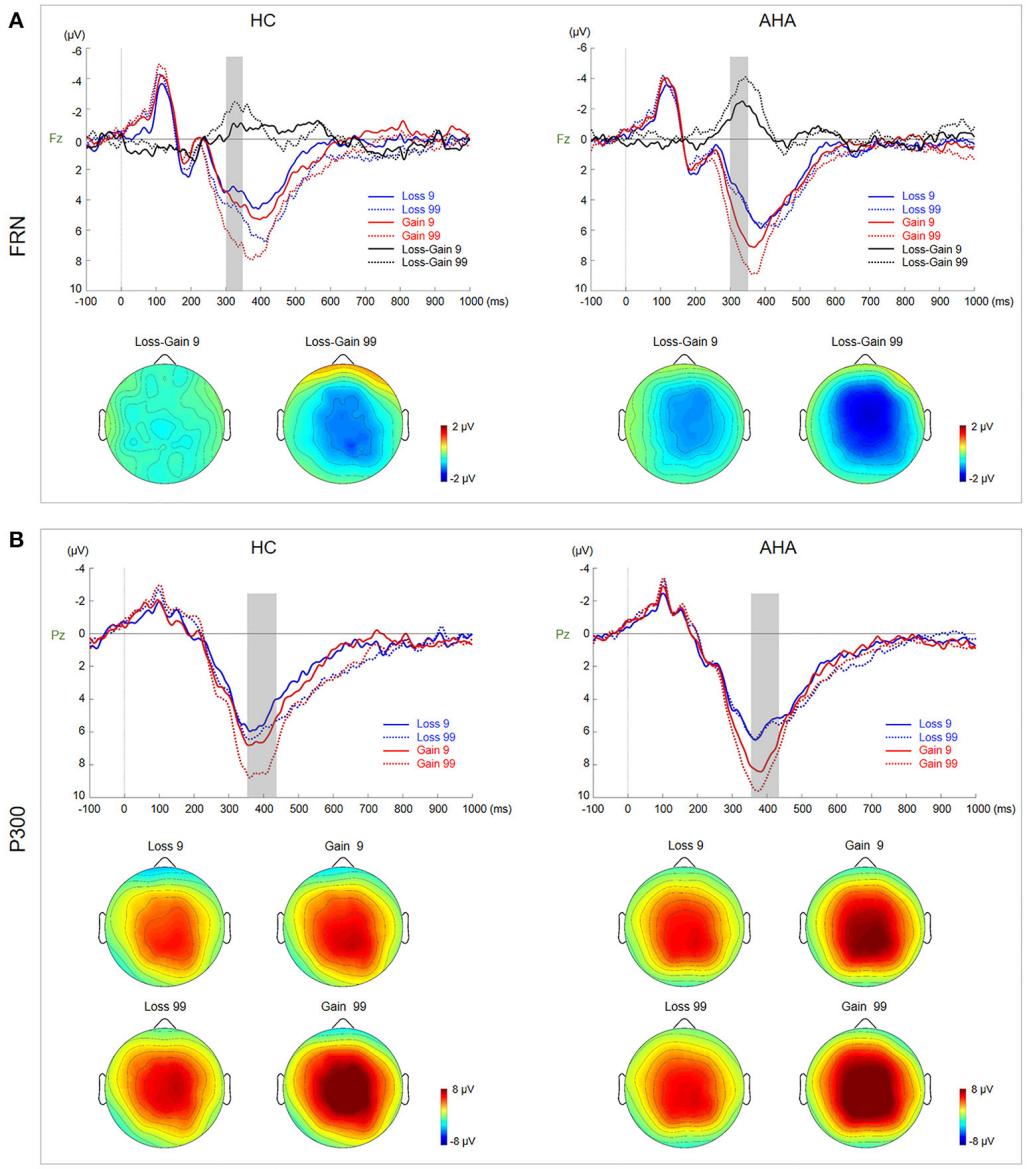
Waveforms and scalp topographies of FRN and P300. **(A)** Grand average ERP waveforms for gain and loss by risk and sensation seeking at Fz. FRN (calculated as the difference between loss and gain waveforms) for low- and high-risk outcomes is shown with shaded areas depicting the time window. Scalp maps (290–350 ms) show the topography of the FRN component averaged across subjects and time windows. **(B)** Grand average ERP waveforms for gain and loss by risk and sensation seeking at Pz; the shaded area depicts the P300 time window. Scalp maps (360–420 ms) show the topography of the mean amplitude of the P300 time window.

According to grand average waveforms and topographic maps (Figures 2, 3), the SPN time window was set from −200 to 0 ms before feedback onset at lateral electrode sites (FT7/8), where SPN has maximal values (Zheng and Liu, 2015). To isolate the FRN component, we obtained difference waveforms for low-risk (loss minus gain following a low-risk choice) and high-risk (loss minus gain following a high-risk choice) outcomes (Holroyd et al., 2009; Walsh and Anderson, 2011), which could minimize the overlap between FRN and the other ERP components. FRN was defined at Fz and FCz with a time window between 290 and 350 ms after feedback onset. P300 was measured by averaged activities across 360 to 420 ms post-feedback onset at CPz and Pz due to a posterior distribution.

### ERP source localization

To identify neural generators involved in risky decision-making and their corresponding functional roles in the decision-making process, the brain sources of each ERP components were reconstructed using a well-established source localization method (Pascualmarqui et al., 2011). The forward head model was built by the boundary element method (BEM), using the MNI152 template (Fuchs et al., 2002) and standard electrode positions of the EEG cap (Montreol Method: 10–20 electrode system). Then, brain activity in each brain voxel was estimated by exact low-resolution brain electromagnetic tomography (eLORETA), which shows lower localization error and clearer visibility compared with LORETA (Jatoi et al., 2014). Specifically, a current density analysis was performed of scalp-recorded electrical activity using the sLORETA and eLORETA software package (Pascual-Marqui, 2002; Pascualmarqui et al., 2011). The resulting eLORETA images represented the electrical neural activity of each voxel in the neuroanatomic Montreal Neurological Institute (MNI) space, as amplitude of the computed current source density (μA/mm^2^). The brain sources were restrained in the cortical gray matter at 5 mm resolution, resulting in 6,239 cortical gray matter voxels in total.

To enhance the spatial sensitivity of ERP components, the following ERP time windows were used in the EEG source analysis: SPN, −100 to 0 ms; FRN, 290 to 350 ms; P300, 360 to 420 ms. Inverse source reconstruction was performed for each experimental condition (options, “9” or “99”; outcomes, “+9,” “−9,” “+99,” “−99”) and group (AHAs, HCs), respectively, resulting in four distinct conditions (gain or loss, high- or low-risk) at the source localization. The estimate sources were normalized for all participants within each group for each condition. It is important to note that given the ill-posed EEG source localization, the maps presented in this study should be considered rough estimates of human brain sources during the gambling task.

### Statistical analysis

For behavior results, independent samples *t*-test was performed for accumulated credits in the task and reaction times (RTs) between AHAs and HCs. A 2 × 2 repeated measures ANOVA was performed with group (AHAs vs. HCs) and risk (9 vs. 99) as between- and within-subjects factors, respectively. Statistical analysis of ERP components was performed with SPSS 19.0 (IBM, Armonk, NY). A test of homogeneity of variances was performed, with F values adjusted with Brown-Forsythe's and Welch's corrections when necessary. For repeated measures ANOVA, Mauchly's test was used to assess sphericity, with the Greenhouse-Geisser correction applied when necessary (Cao et al., 2017).

To evaluate the characteristics of decision-making in AHAs, ERP components were compared between AHAs and HCs. In particular, we examined the SPN component at the anticipation stage, and FRN and P300 at the outcome-evaluation stage. For the SPN component, a 2 × 2 × 2 repeated measures ANOVA was performed with group (AHAs vs. HCs) as a between-subjects factor, and risk (9 vs. 99) and hemisphere (left vs. right) as within-subjects factors. For the FRN component, a 2 × 2 repeated measures ANOVA was performed with group (AHA vs. HC) and difference waveforms in amplitude (loss-gain 9/99) as between- and within-subjects factors, respectively. 2 × 2 × 2 repeated measures ANOVA was also performed with group (AHA vs. HC) as a between-subjects factor, and risk (9 vs. 99) and valence (gain vs. loss) as within-subjects factors for the P300 amplitude.

We further compared brain sources between AHAs and HCs in the same condition (between-subjects comparison) or different conditions in the same group (within-subjects comparison). Group-level source images were generated with group as a between-subjects factor in each condition. *F*-test was performed to assess the effect per time window of each ERP component, and per condition within each group (AHAs or HCs) and between groups (AHAs vs. HCs). Family-wise error (FWE) corrected *P* < 0.05 was considered statistically significant. In addition, voxel-wise *t*-test (two-tailed) was performed to compare current density in each condition and group, as well as between groups.

## Results

### Behavioral performance

Independent samples *t*-test revealed that HCs and AHAs show no significant difference in terms of accumulated final credits in the task [*t*_(38)_ = 0.324, *p* = 0.748; AHA, *M* = 243.0, *SE* = 199.2; HC, *M* = 172.5, *SE* = 158.0]. ANOVA showed no interaction effect [*F*_(1, 38)_ = 0.222, *p* = 0.640] between group and the decision-making time (defined by reaction time, RT), and no main between group effect [*F*_(1, 38)_ = 0.172, *p* = 0.640]. In addition, high-risk choice proportion (times of high risky choices in all 400 trials) had a significant group effect [*F*_(1, 38)_ = 13.48, *p* = 0.001; AHA, 0.7125 ± 0.0667; HC, 5601 ± 0.1204], indicating more high-risk choices in AHAs compared with HCs. Besides, there was a significant interaction effect between group and risky option proportion [*F*_(1, 38)_ = 6.717, *p* = 0.013]. Further simple and simple effect test showed a significant difference in the high-risk condition between the two groups [*F*_(1, 38)_ = 24.62, *p* < 0.001] with a tendency for higher-risk in AHAs compared with HCs (AHAs, 0.7125 ± 0.0667; HCs, 0.5601 ± 0.1204).

### SPN: outcome expectation stage

A significant main group effect [*F*_(1, 38)_ = 5.045, *p* = 0.031] and a significant interaction effect between group and hemisphere [*F*_(1, 38)_ = 6.670, *p* = 0.014] were observed in the SPN component. Further simple effects test found no significant difference in left hemisphere activity [*F*_(1, 38)_ = 0.80, *p* = 0.377] while right hemisphere activity showed a significant difference between the two groups [*F*_(1, 38)_ = 7.67, *p* = 0.009]. Interestingly, the mean amplitude of SPN in the right hemisphere was higher in AHAs than HCs (Figures 2A,B). Source localization results showed that the middle frontal gyrus (MFG), ACC, postcentral gyrus, insular for low-risk option, and insular for high-risk options were significantly more deactivated in AHAs than in HCs (Figure 2C).

### FRN and P300: outcome evaluation stage

Topographic maps for AHAs and HCs depicting voltage differences across the scalp for FRN from 290 to 350 ms after feedback onset are presented in Figure 3A. Grand average ERP waveforms at Fz elicited by gain and loss as well as difference waveforms (loss minus gain) are also presented in Figure 3A. The amplitude of difference waveforms of FRN had a significant main group effect [*F*_(1, 38)_ = 6.806, *p* = 0.013], indicating that more negative FRN was elicited for AHAs vs. HCs (AHAs: *M* = −2.664, *SE* = 0.417; HCs: *M* = −1.125, *SE* = 0.417]. There was a significant main effect of risk (low- vs. high-risk) [*F*_(1, 38)_ = 9.659, *p* = 0.004], suggesting that high risk options (*M* = −2.556, *SE* = 0.392) evoked more negative FRN compared with low risk counterparts (*M* = −1.233, *SE* = 0.333). However, no significant interaction was found for group risk [*F*_(1, 38)_ = 0.000; *p* = 0.987].

**Figure 4:**
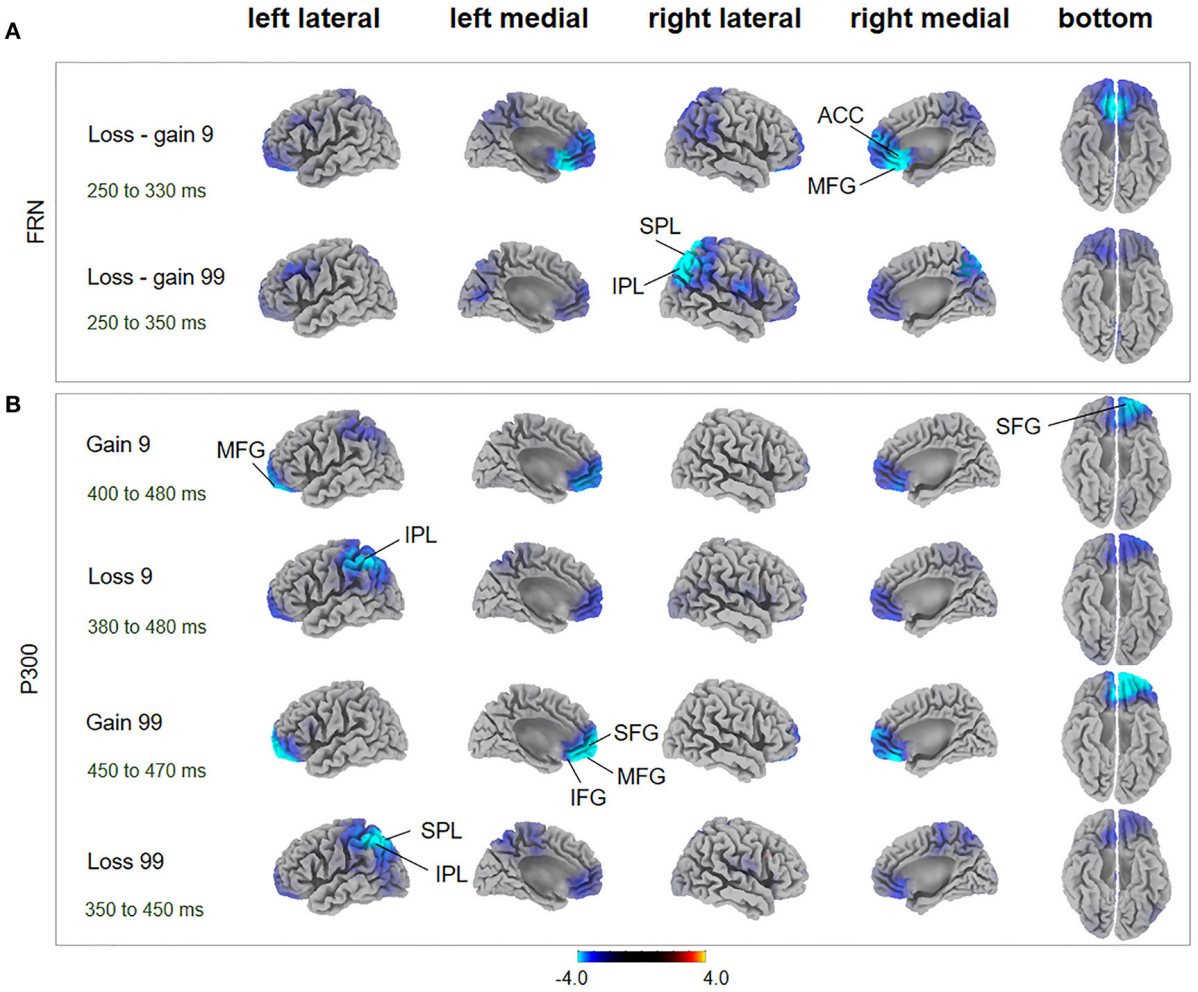
FRN and P300 source localization and between-subject comparison. The *t*-score maps for comparisons between AHAs and HCs for the FRN **(A)** and P300 **(B)** components are shown for low- and high-risk options, as well as feedback outcomes, respectively. The brain regions labeled with yellow/red indicate AHAs > HCs, while green/blue indicates AHAs < HCs. The time periods for different components are also indicated. Significant brain regions (*p* < 0.05, FWE corrected) are indicated on the map. The peak MNI coordinate regions for each comparison are reported in Table 1. ACC, anterior cingulate cortex; IPL, inferior parietal lobule; IFG, inferior frontal gyrus; MFG, middle frontal gyrus; SPL, superior parietal lobule; SFG, superior frontal gyrus.

During the FRN time window, in the loss-gain 9 condition, difference of sources were mainly identified in the ACC, while in the loss-gain 99 condition differences were located in the inferior parietal lobule (IPL) and superior parietal lobule (SPL) between the two groups (Figure 4A, Table 1). We found that activities in the ACC and parietal areas were significantly reduced in AHAs compared with HCs.

**Table 1:** Results of between-subjects factor comparisons for ERP sources.

**ERPs**	**Time (ms)**	**Condition**	**Coordinate region**	**Peak MNI coordinate**			***P*-value**
SPN	−100–0	Select 9	MFG	−25	−10	45	0.0016^**^
			ACC	−20	−20	40	
			Right insula	40	−25	20	
			Postcentral gyrus	−30	−20	45	
		Select 99	Right insula	45	−25	20	0.0496^*^
FRN	290–350	Loss-gain 9	ACC	5	30	−10	0.0140^*^
			MFG	5	30	−15	
		Loss-gain 99	SPL	−35	−70	50	0.0070^**^
			IPL	30	−60	45	
P300	360–420	Gain 9	SFG	−25	60	−15	0.0404^*^
			MFG	−20	55	−10	
		Loss 9	IPL	−50	−50	50	0.0486^*^
		Gain 99	SFG	−30	60	0	0.0090^**^
			ACC	−5	40	−10	
			IFG	−10	40	−20	
			Orbital gyrus	−5	40	−20	
		Loss 99	SPL	−40	−60	50	0.0388^*^
			IPL	−40	−55	50	

Peak MNI coordinates for between-subjects factor comparison between AHAs and HCs. Clusters surviving FWE-corrected threshold p < 0.05. ACC, anterior cingulate cortex; IPL, inferior parietal lobule; IFG, inferior frontal gyrus; MFG, middle frontal gyrus; SPL, superior parietal lobule; SFG, superior frontal gyrus.

* p < 0.05;

** *p < 0.01*.

Grand average ERP waveforms at Pz elicited by gain and loss are presented in Figure 4B. Topographic maps for P300, from 360 to 420 ms, are also shown in Figure 4B. There was a significant interaction between risk and valence (gain vs. loss) [*F*_(1, 38)_ = 11.230, *p* = 0.002]. Further simple effects test showed a larger P300 in high risk (*M* = 8.704, *SE* = 0.632) than low risk (*M* = 7.383, *SE* = 0.565) choices for the gain condition, but not for the loss condition (high risk: *M* = 6.023, *SE* = 0.487; low risk: *M* = 5.989, *SE* = 0.494).

During the P300 time window, significant voxel clusters of differential activation between the two groups were found in the superior frontal gyrus (SFG) and MFG for the gain 9 condition, in the IPL for the loss 9 condition, in the SFG, MFG, ACC, inferior frontal gyrus (IFG), and orbital gyrus for the gain 99 condition, and in the SPL and IPL for the loss 99 condition (Figure 4B, Table 1). Generally, prefrontal cortex activity decreased overtly in AHAs but not in HCs. In this study, we mainly focused on between groups differences; additional results of within-subjects comparisons are shown in Figure S1 and Table S1.

## Discussion

This study assessed the behavioral and neural characteristics of risky decision-making in AHAs, in a simple gambling task. We expected behavioral and neural response abnormalities of risky decisions in AHAs. Despite no significant differences in final scores and decision-making times between the AHA and HC groups, behavioral results showed that AHAs tend to select more high-risk options compared with HCs in the gambling task, confirming our hypothesis that AHAs are more risk-seeking compared with HCs. This corroborates previous reports that drug addicts have a high rate of risk-taking behaviors (Brand et al., 2006). Such findings are consistent with existing studies showing AHAs are insensitive to risk under risky situations (Brand et al., 2006), and are persistently engaged in risky behaviors even after losing considerable scores while failing to adjust to a safer strategy to avoid risk (Cheng et al., 2015). Similar results have been reported for internet addiction (Ko et al., 2010) and alcoholism (Bjork et al., 2004; Cservenka and Nagel, 2012).

ERP components could be potential electrophysiological factors involved in various processing stages of the gambling task, including the reward anticipation (indexed by SPN) and outcome-evaluation (indexed by FRN and P300) stages (Zheng and Liu, 2015). The SPN amplitude was characterized by right hemisphere dominance in HCs (Figure 2B), in line with previous studies (Kotani et al., 2009; Yasunori et al., 2015). However, such right hemisphere dominance of SPN disappeared in AHAs. Consistently, source localization showed that brain activities in AHAs in the SPN time window were significantly reduced in the right insula and ACC (Figure 2B), which are regions involved in risk-taking decision making (Lee et al., 2009). Given the right anterior insula does underlie SPN right hemisphere preponderance (Kotani et al., 2009), deactivated right insula in AHAs may result in the disruption of SPN right hemisphere preponderance. Besides, Yan et al. (2014), assume that the insula could integrate interoception states into conscious feelings and subsequently into decision-making processes that are involved in uncertain risk and reward. Accumulative evidence indicates that insula activity reflects the risk prediction error (Kuhnen and Knutson, 2005). Therefore, abnormal insula activities may impair daily life decision-making and performance on gambling tasks (Dunn et al., 2006). Previous studies have demonstrated that SPN could reflect affection and motivation in outcome anticipation (Kotani et al., 2010; Ohgami et al., 2010) and indicates anticipation of receiving a reward feedback (Zheng and Liu, 2015). The disappearance of right dominance in SPN and deactivated right insula demonstrated the abnormal cognitive process in risk evaluation for the AHA group. The insula is commonly activated in uncertain situations, which fits with the reward anticipation stage in the present task. Abnormal SPN and insula activation might affect outcome anticipation in AHAs and further influence the adjustment of the selection strategy for next trials based on the outcome of the current trial. Moreover, previous studies have identified two key ERP components related to outcome evaluation, including FRN and P300 (Nieuwenhuis et al., 2004; Pedroni et al., 2011; Osinsky et al., 2014). In this study, the FRN amplitude, defined as the difference between loss and gain (Qu et al., 2013), was enhanced in high-risk conditions compared with low-risk conditions in both groups (Figure 3A). More importantly, we found enhanced overall FRN in AHAs compared with HCs in both conditions. According to previous studies, FRN is associated with negative feedback and unfavorable outcome, such as incorrect response or monetary loss, rather than positive outcome (Miltner et al., 1997; Müller et al., 2005). In addition, FRN reflects the early appraisal of feedback on a binary classification basis of positive vs. negative outcomes (Hajcak et al., 2006). Greater deviation of expectation and actual outcomes leads to larger FRN (Cao et al., 2015). Thus, high FRN amplitude in AHAs may result from unpredicted positive outcomes. Another possible explanation for higher FRN in AHAs is a high motivation for high-risk options in these individuals (Luo et al., 2011). In the FRN time window, sources were mainly identified in the ACC, MFG, SPL, and IPL (Figure 4A, Table 1). Previous studies have shown that the ACC is involved in the assessment of motivational impact of outcome events, but not in evaluating performance (Gehring and Willoughby, 2002). The SPL and IPL might be more involved in reward processing (Liljeholm et al., 2011). In addition, these regions are associated with the reflective neural system of the prefrontal cortex for decision-making (Asp et al., 2013), forecasting the future consequences of a behavior, and inhibitory control. Abnormal activities in these regions might result in an abnormal reflective system so that AHAs could not adjust their decision strategies, eventually leading to risk-taking behaviors without subjective control.

At the later cognitive appraisal stage, P300 was larger for high-risk outcomes and gain trials (Figure 3B), indicating that P300 is sensitive to both risk magnitude (low- or high-risk) and valence (gain vs. loss) (Sato et al., 2005). Our results showed that positive outcomes [P300 amplitude (gain > loss)] could often induce larger P300 (Hajcak et al., 2007, 2010). In addition, risk magnitude significantly affected the P300 amplitude in difference valence and group conditions, indicating that P300 may reflect the depth processing of positive and negative rewards (Zheng and Liu, 2015). A main group effect was not found in this study, indicating that the AHA and HC groups might have similar cognitive processes in late outcome evaluation. Besides, P300 may reflect a later, top-down controlled feedback evaluation process, in which factors related to the allocation of attentional resources (Cui et al., 2013), including valence, magnitude expectancy, and risk magnitude (Sato et al., 2005; Wu and Zhou, 2009) play important roles and are likely related to high-level motivational and affective evaluation (Zhou et al., 2010). Although we did not observe a group effect on the P300 amplitude, the identified brain areas with differences at the source level between the two groups were mainly in the SFG, MFG, ACC, SPL, and IPL (Figure 4A, Table 1). The IPL and MFG are associated with response selection, cross-trial performance monitoring, and working memory processes (Elliott et al., 1997; Hosseini et al., 2010). The SPL is involved in the cognitive process during decision-making (Wallentin et al., 2015), such as reward magnitude processing. The ACC is involved in learning from monetary feedback, encoding of negative feedback (Kringelbach and Rolls, 2003; Bellebaum et al., 2008), and integrating information about outcome valence and magnitude (Knutson and Cooper, 2005). More importantly, the above regions are also associated with the reflection system for decision-making. Abnormal function of the reflective prefrontal cortex could lead to impaired response inhibition and abnormal salience attribution in addiction (Spitzer, 1980), which provides an explanation of why AHAs have a weakened impulse-control system in the pursuit of immediate pleasure at the expense of long-term consequences. Besides, an abnormal reflective system may impact emotion and/or memory, compromising the ability to make advantageous decisions (Bechara et al., 1997).

Although this study revealed the characteristics of different decision-making stages in AHAs compared to HCs from the ERP and source localization perspectives, it remains unclear how different brain regions interact with each other during decision-making. Moreover, different cognitive states during risk decision-making were assessed in this study, but whether a specific brain area plays a key role in the entire decision-making process was not evaluated. We did not measure the level of the stress or anxiety during/after experiment, which largely prevented us from linking the behavioral or neural response with the psychological states of subjects. In addition, low-density EEG montage and the use of volume conduction head model template in this study might limit the spatial resolution and accuracy of source reconstruction (Liu et al., 2016), thus the interpretation of our source localization results should be more cautious. Finally, that the reflective system weakens inhibition control and the insula abnormally integrates viscerosensory information may be important reasons explaining abnormal decision-making in AHAs.

## Conclusion

Our findings revealed significant differences in ERPs and corresponding neural sources between AHAs and HCs during the decision-making process; these differences are reflected in the anticipation and outcome-appraisal stages. We assume that such differences are probably caused by an abnormal reflective system due to decreased activation in the right anterior insula and MFG. This study provides electrophysiological evidences for understanding the neural mechanisms of abnormal decision-making behaviors in heroin dependence, and has the potential to offer novel biomarkers for withdrawal treatment in addicts.

## Author contributions

Study conception and design: BH and QL. Acquisition of data: QZ and HL. Analysis and interpretation of data: HL, BH, HW, and QL. Manuscript writing: All authors.

## Conflict of interest statement

The authors declare that the research was conducted in the absence of any commercial or financial relationships that could be construed as a potential conflict of interest.
