# Supplementary material for: Abstinent Heroin Addicts Tend to Take Risks: ERP and Source Localization
Source: Front Neurosci. 2017 Dec 6;11:681. doi: 10.3389/fnins.2017.00681 (PMC5723666; doi:10.3389/fnins.2017.00681)
Supplement: Supplementary file 1 [file DataSheet1.pdf]

Supplementary Material

Abstinent heroin addicts tend to take risks: ERP and source localization

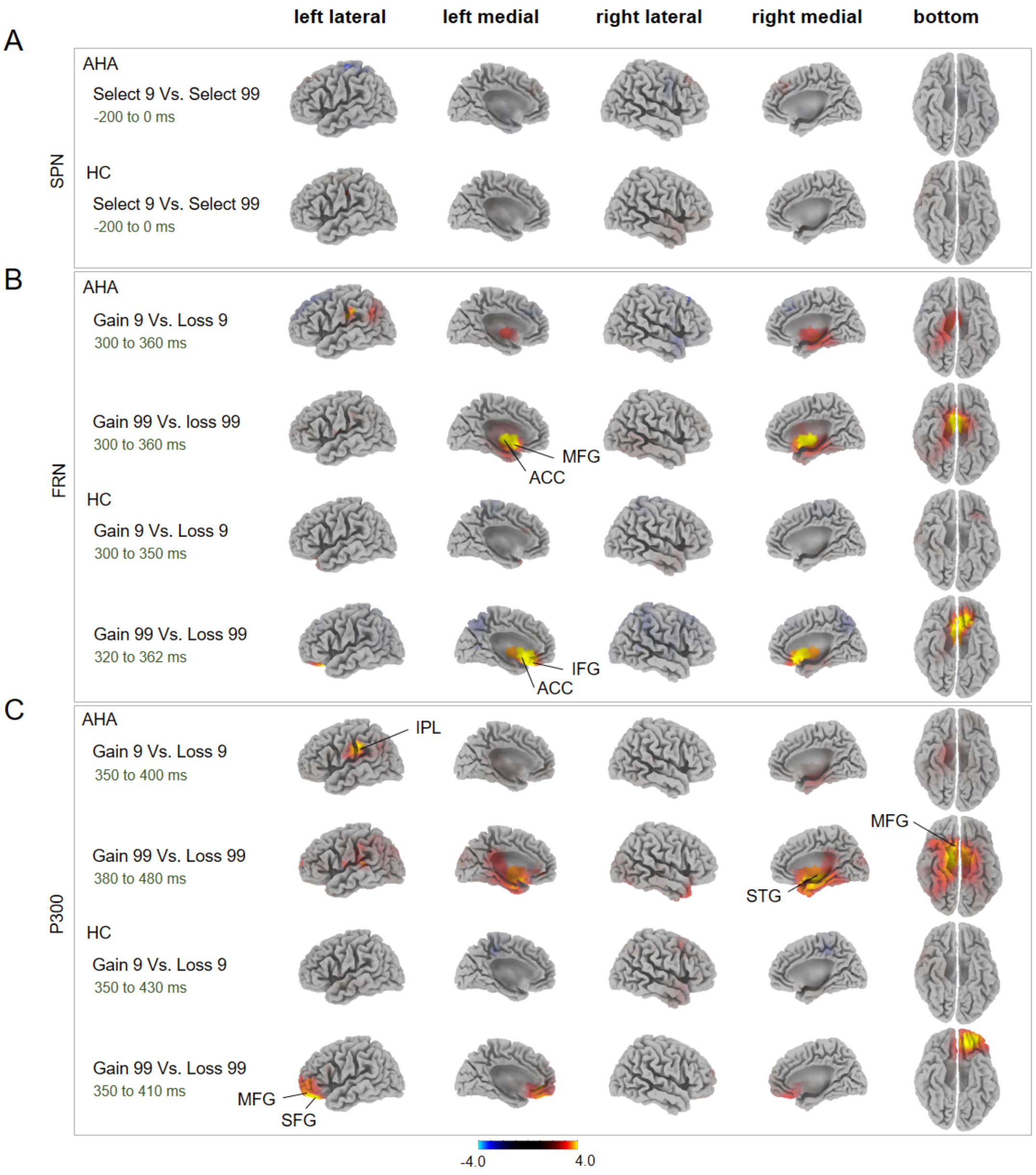

Figure S1 –Within-group comparisons for SPN, FRN and P300 sources. The t-score maps for within-subjects comparison are shown in low- (select 9) and high-risk (select 99) options for the SPN (up panel, A), FRN (middle panel, B), and P300 (bottom panel, C) of feedback outcomes,

respectively. The brain regions labeled with yellow/red indicate increased activity of the former compared with the latter (e.g. gain vs. loss). Significant brain regions (p<0.05, FWE corrected) are indicated on the map. The peak MNI coordinates for the contrast per condition in each group are described in ***Supplementary Table S1***. ***Abbreviations:*** *ACC, anterior cingulate cortex; IPL, inferior parietal lobule; IFG, inferior frontal gyrus; MFG, middle frontal gyrus; SFG, superior frontal gyrus; STG, superior temporal gyrus.*

Supplementary Table S1 - Within-subjects comparisons for various ERP sources.

ERPs	Time (ms)	Condition	Coordinate region	Peak MNI Coordinate			P-value
			**AHA**				
SPN	-100 - 0	Select 9 Vs. Select 99					0.1502
FRN	290 - 350	Gain 9 Vs. Gain 99	Postcentral gyrus	-50	-25	30	
		Loss 9 Vs. Loss 99					0.3944
		Gain 9 Vs. Loss 9	Postcentral gyrus	-50	-25	30	0.0364*
		Gain 99 Vs. Loss 99	ACC	5	5	-5	0.0008**
			MFG	15	10	-20	
			Hippocampus	15	-5	-15	
P300	360-420	Gain 9 Vs. Gain 99	IPL	-60	-25	35	0.0152*
			Postcentral gyrus	-55	-25	30	
		Loss 9 Vs. Loss 99					0.2142
		Gain 9 Vs. Loss 9	IPL	-55	-25	30	0.0146*
			Postcentral gyrus	-60	-25	35	
		Gain 99 Vs. Loss 99	MFG	-15	45	5	0.0046**
			STG	-50	-30	15	
			**HC**				
SPN	-100 - 0	Select 9 Vs. Select 99					0.1832
FRN	290 - 350	Gain 9 Vs. Gain 99	MFG	15	-15	60	0.0358*
			SFG	20	-15	70	
		Loss 9 Vs. Loss 99					0.2392
		Gain 9 Vs. Loss 9					0.7994
		Gain 99 Vs. Loss 99	IFG	-20	30	-5	0.0036**
			ACC	-5	20	-5	
P300	360-420	Gain 9 Vs. Gain 99	MFG	10	-15	60	0.0284*
		Loss 9 Vs. Loss 99					0.4406
		Gain 9 Vs. Loss 9					0.5440
		Gain 99 Vs. Loss 99	SFG	-15	50	-20	0.0090**
			MFG	-15	45	-20	
			Orbital gyrus	-10	50	-25	

Peak MNI coordinates for ***between-subjects comparison between AHAs and HCs. Clusters surviving FWE-corrected threshold p<0.05.***

Abbreviations: *ACC, anterior cingulate cortex; IPL, inferior parietal lobule; IFG, inferior frontal gyrus; MFG, middle frontal gyrus; SFG, superior frontal gyrus; STG, superior temporal gyrus.*
